# Therapeutic potential of targeting macrophage polarization in metastatic gastric cancer: a review on core mechanisms and clinical progress

**DOI:** 10.1007/s12094-026-04370-0

**Published:** 2026-06-04

**Authors:** Ningning Gong, Guanmin Qin, Junxia Sun, Shiying Shen, Xinxing Zhang, Minmin Lu

**Affiliations:** 1 Weifang Traditional Chinese Hospital, Weifang, 261041 Shandong China; 2 Sunshine Union Hospital, Weifang, 261000 Shandong China

**Keywords:** Gastric cancer metastasis, Tumor-associated macrophages, Macrophage polarization, Metabolic reprogramming, Immune checkpoint inhibitors (ICIs), Exosomal non-coding RNAs

## Abstract

Gastric cancer remains a leading cause of cancer-related mortality worldwide, with high metastatic potential being a critical factor for poor prognosis. Notably, the objective response rate (ORR) of current immunotherapies, such as immune checkpoint inhibitors (ICIs), remains below 20%. Within the tumor microenvironment (TME), the dynamic interactions among immune cells profoundly regulate tumor progression, in which the remarkable plasticity of tumor-associated macrophages (TAMs) plays a pivotal role. Under the induction of microenvironmental signals, TAMs can polarize into either the anti-tumor M1 phenotype or the pro-tumor M2 phenotype. This review summarizes the research progress of macrophage polarization in distant metastasis of gastric cancer, with a specific focus on the regulatory mechanisms of core signaling pathways, including STAT3, PI3K/AKT, and exosomal ncRNA regulatory networks, in macrophage functional reprogramming and metastatic niche formation. Furthermore, we discuss potential immunotherapy strategies centered on modulating macrophage phenotypes, such as CSF1R inhibitors, CD47 blockers, and their combination with ICIs. These findings provide a significant foundation for developing macrophage-targeted therapeutic interventions, screening translational biomarkers, and improving the prognosis of patients with metastatic gastric cancer.

## Introduction

Gastric cancer (GC) ranks as the fifth most common malignancy and the fourth leading cause of cancer-related mortality worldwide. According to the 2024 global cancer statistics from the International Agency for Research on Cancer (IARC), annual new cases exceed 1 million, with more than 650,000 deaths [1]. Due to the insidious onset of early symptoms, approximately 70% of patients are diagnosed at an advanced stage or with distant metastasis, resulting in a 5-year survival rate of less than 30% [2]. The burden of GC is particularly prominent in China, where about 70% of cases are advanced or metastatic (primarily to the liver, lungs, and peritoneum) at diagnosis, with a survival rate consistently below 30% [3]. Despite advances in multimodal therapies—including surgery, targeted therapy, and immune checkpoint inhibitors—the objective response rate for metastatic GC remains under 20% [4]. A core bottleneck lies in the insufficient understanding of the mechanisms underlying immune evasion and suppression within the tumor microenvironment (TME) during metastasis formation, particularly the immune suppression mediated by tumor-associated macrophages (TAMs).

Tumor metastasis depends not only on the intrinsic malignant properties of tumor cells but also on the composition and dynamic interactions of immune cells. As the most abundant immune cell type in the TME, macrophages exhibit high polarization plasticity (pro-inflammatory/anti-tumor M1 and anti-inflammatory/pro-tumor M2), which directly dictates the orientation of the immune microenvironment [4, 5]. Clinical studies have confirmed that tumor-associated macrophages (TAMs) in GC tissue are predominantly of the M2 phenotype, and their infiltration density is positively correlated with the risk of distant metastasis [6]. This review aims to systematically integrate the critical roles of macrophage polarization, signaling pathways, and immune microenvironment remodeling mechanisms in the distant metastasis of gastric cancer, with a particular focus on summarizing clinical translational strategies targeting TAMs (including drug targets and combination therapies). It is intended to provide guidance for clinical trial design and biomarker development for precision immunotherapy in gastric cancer.

## Methods

This study is presented as a narrative review focused on integrating mechanistic research with clinical evidence. Comprehensive searches were performed across databases including PubMed, Web of Science, Embase, and Google Scholar, with a coverage period ending in October 2025. The search strategy combined MeSH terms with free-text words, utilizing keywords such as “gastric cancer distant metastasis”, “macrophage polarization”, “M1/M2 macrophage”, and “tumor microenvironment” connected via AND/OR Boolean operators to refine the results. Reference lists of the included studies were manually cross-referenced to identify any potentially missed relevant literature.

The inclusion criteria were: (1) direct relevance to gastric cancer distant metastasis and macrophage polarization; (2) provision of experimental data at the molecular, cellular, animal, or clinical levels; and (3) a clear study design with reliable results. Exclusion criteria consisted of conference abstracts, studies without empirical data, and duplicate publications. This review adopts a narrative approach focused on integrating mechanistic research with clinical evidence, primarily analyzing the role of macrophage polarization in gastric cancer metastasis, the signaling pathway networks, and the corresponding therapeutic potential.

## Theoretical foundations of macrophage polarization and gastric cancer distant metastasis

### Classification and functional heterogeneity of macrophage polarization

As critical regulators of innate and adaptive immunity, macrophages originate from bone marrow-derived monocytes and possess significant phenotypic plasticity [7]. Under distinct microenvironmental stimuli, they can differentiate into two primary functional subtypes: the classical activation pathway (M1 polarization) and the alternative activation pathway (M2 polarization) [8]. In the classical activation pathway, macrophages are stimulated by pro-inflammatory signals such as bacterial lipopolysaccharide (LPS) and interferon-gamma (IFN-γ) [9]. Upon activation via the nuclear factor-kappa B (NF-κB) and mitogen-activated protein kinase (MAPK) pathways, macrophages highly express markers including inducible nitric oxide synthase (iNOS), major histocompatibility complex class II (MHC II), and costimulatory molecules such as CD80 and CD86. Simultaneously, they secrete pro-inflammatory cytokines, including tumor necrosis factor-alpha (TNF-α), interleukin-1β (IL-1β), IL-6, IL-12, and IL-23, thereby enhancing antigen presentation and promoting cytotoxic immune responses [10, 11].

In the alternative activation pathway, macrophages are induced by anti-inflammatory signals, including interleukin-4 (IL-4), IL-13, IL-10, and transforming growth factor-beta (TGF-β) [12]. These cells express specific markers such as arginase-1 (Arg1), mannose receptor (CD206), and CD163, and secrete immunosuppressive factors including IL-10 and TGF-β. Functional roles of this subset encompass tissue repair, angiogenesis, and the promotion of tumor immune evasion [13].

In fact, macrophage polarization is not a static M1/M2 binary process but rather a dynamic functional continuum continuously shaped by microenvironmental signals. Beyond the classical M1 and M2 subtypes, a diverse array of macrophage subpopulations exists in vivo, including intermediate transition, tissue-resident, metabolically adapted, and tumor-microenvironment-specialized clusters, each exhibiting distinct induction signals, molecular signatures, and biological functions. However, all these subpopulations essentially represent context-dependent states along the M1–M2 polarization spectrum rather than independent lineages. Intermediate transition macrophages respond to concurrent pro-inflammatory and anti-inflammatory stimuli, co-expressing M1 and M2 marker genes and secreting cytokines such as IL-6 and IL-10, which possess dual roles in inflammatory regulation and immunosuppression to maintain local immune homeostasis [14]. Tissue-resident macrophages are induced by tissue-specific niche signals and are characterized by high expression of CD163, CD209, and MerTK; these cells primarily secrete TGF-β and IL-10 to facilitate tissue maintenance and injury repair [15]. Metabolically adapted macrophages are activated by metabolic cues such as lactate and hypoxia and are characterized by high expression of HIF-2α, PPARγ, and GPR81; they secrete VEGF and MMP9 to mediate metabolic reprogramming and angiogenesis [16]. Specialized macrophages within the tumor and metastatic niche undergo directional differentiation under the influence of microenvironmental factors such as CSF-1, TGF-β, and CCL2. These subsets characteristically overexpress SPP1, TREM2, C1QC, and APOE, while secreting IL-10, TGF-β, CCL2, and VEGF to drive tumor progression and immunotherapy resistance by reinforcing immunosuppression, mediating metabolic symbiosis, and promoting matrix remodeling and metastatic colonization [17].

As M1/M2 polarization represents the core functional determinant governing macrophage pro-tumor and anti-tumor roles in gastric cancer progression and distant metastasis, this review adopts the M1/M2 paradigm as its central framework to depict the full plasticity and functional spectrum of macrophages. Accordingly, key mechanisms, signaling regulation, and therapeutic strategies are systematically elucidated within this core M1/M2 axis.

### Mechanisms and pathways of gastric cancer distant metastasis

Distant metastasis is the primary cause of poor prognosis in gastric cancer (GC) patients, characterized by significant organ specificity and multifactorial driving mechanisms. Clinically, the liver, lungs, peritoneum, and distant lymph nodes are the most common metastatic sites. Among these, the liver serves as the predominant target organ for hematogenous metastasis due to the anatomical characteristics of gastric venous blood flowing through the portal venous system [18]. Approximately 30%–50% of patients with advanced GC develop liver metastasis, and their 5-year survival rate is significantly lower than that of patients without metastasis [19]. Although pulmonary and peritoneal metastases are relatively less frequent, they often lead to severe complications such as ascites and intestinal obstruction, markedly reducing quality of life. Beyond anatomical factors, tumor stage, degree of differentiation, and pathological types (especially poorly differentiated adenocarcinoma and signet ring cell carcinoma) significantly influence the risk of distant metastasis. As research progresses, increasing evidence suggests that the holistic remodeling of the tumor microenvironment (TME), rather than changes in a single cell population, serves as the intrinsic foundation for metastatic occurrence [20, 21].

At the molecular level, gastric cancer (GC) distant metastasis is a multi-step process driven by sequential or parallel biological events. The epithelial–mesenchymal transition (EMT) is considered a critical stage for tumor cells to acquire migratory and invasive properties [22]. The TGF-β/Smad signaling pathway holds a central position in EMT by upregulating transcription factors such as Snail, Slug, and Twist. This suppresses the epithelial marker E-cadherin while promoting the expression of mesenchymal markers, including Vimentin and N-cadherin, thereby enhancing cellular motility [23]. The Wnt/β-catenin and Notch pathways synergize with TGF-β to activate the EMT program, maintaining the mesenchymal phenotype and stemness characteristics [24]. Subsequently, tumor cells degrade the extracellular matrix (ECM) by secreting matrix metalloproteinases (MMPs), specifically MMP2 and MMP9, which disrupt the basement membrane structure and enable entry into the circulatory system [25].

Tumor angiogenesis represents another pivotal link in GC distant metastasis. Tumor growth and metastasis rely on an adequate blood supply, which provides nutrients and oxygen while creating channels for cancer cell intravasation. Vascular endothelial growth factor (VEGF) is the key regulator of tumor angiogenesis. GC cells secrete substantial amounts of VEGF, which binds to its receptor (VEGFR) to activate downstream pathways, promoting the proliferation, migration, and lumen formation of vascular endothelial cells [26]. Furthermore, VEGF increases vascular permeability, facilitating cancer cell entry into the blood circulation and promoting distant metastasis. In addition to VEGF, factors such as fibroblast growth factor (FGF) and platelet-derived growth factor (PDGF) participate in regulating tumor angiogenesis. Finally, the functional state of immune cells within the tumor microenvironment profoundly impacts GC metastasis; specifically, M1-type macrophages exert anti-tumor effects, whereas M2-type macrophages promote angiogenesis and tumor metastasis [27].

Gastric cancer distant metastasis is a complex process involving multiple molecular biological mechanisms. Factors such as epithelial–mesenchymal transition (EMT), tumor angiogenesis, and the tumor microenvironment interact and synergistically promote the distant metastasis of gastric cancer.

## Signaling pathways of macrophage polarization and their cross-regulatory mechanisms

Macrophage polarization is a core element of immune microenvironment remodeling in gastric cancer, and its phenotypic transition is finely regulated by multiple signaling pathways. These pathways not only determine the functional state of macrophages but also form a complex immunosuppressive network, making them critical targets that hinder cancer immunotherapy. Systematically resolving these signaling mechanisms is of great significance for revealing the molecular basis of immune evasion and targeted intervention in gastric cancer. The various mechanisms by which M2-type macrophages promote tumor progression in the gastric cancer microenvironment are shown in Fig. 1.

**Fig. 1:**
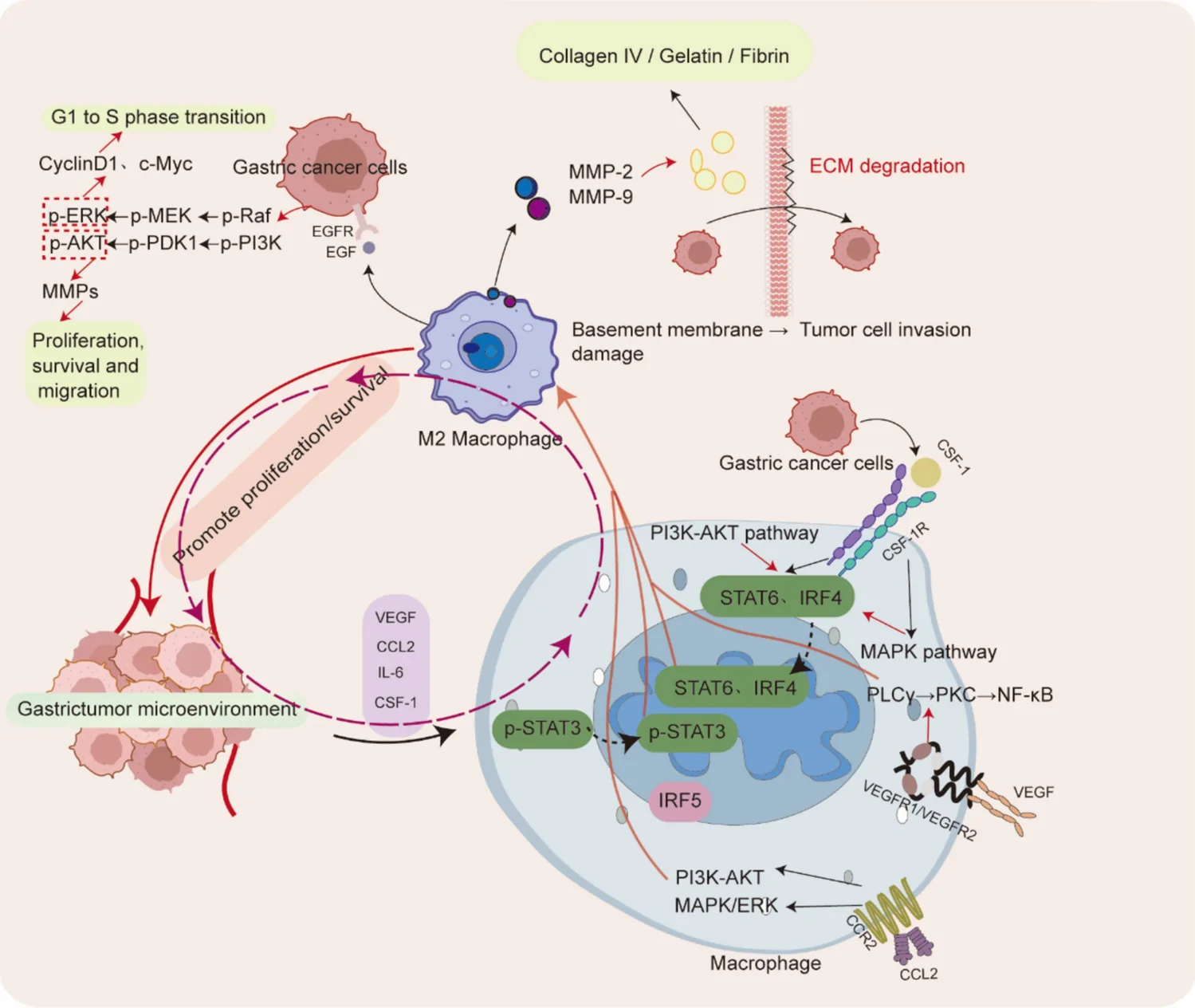
Multifaceted mechanisms by which M2 macrophages promote tumor progression within the gastric cancer microenvironment

### Independent regulatory mechanisms of major signaling pathways

The STAT signaling pathway serves as a pivotal molecular axis regulating macrophage polarization. STAT1 is typically phosphorylated in response to interferon-gamma (IFN-γ) stimulation, promoting the expression of M1-associated genes (e.g., IL-12, TNF-α, iNOS) and thereby maintaining anti-tumor activity. Conversely, constitutive activation of STAT3 induces the upregulation of immunosuppressive cytokines such as IL-10 and TGF-β, skewing macrophages toward the M2 phenotype and promoting angiogenesis and tissue repair [28]. Multiple studies have confirmed that the constitutive activation of STAT3 in gastric cancer tissues is closely correlated with tumor stage, metastasis, and immune tolerance. STAT3-mediated M2 polarization depends on the activation of IL-6/CSF-1 signaling, which provides a mechanistic basis for the clinical application of CSF-1R inhibitors (such as Pexidartinib) in the context of gastric cancer metastasis. This suggests that such inhibitors may reverse the M2 phenotype by blocking the STAT3 pathway [29], thereby possessing significant clinical translational value.

The NF-κB pathway serves as a critical transcriptional regulatory system governing inflammation and immune responses. The canonical NF-κB complex (p65/p50) is released following IκB phosphorylation, subsequently translocating to the nucleus to induce the expression of various inflammatory cytokines. Transient NF-κB activation facilitates the M1-type immune response, whereas sustained or low-level activation favors M2 polarization. This "intensity-dependent effect" constitutes a key mechanism driving the transition from chronic inflammation to immunosuppression in gastric cancer [30].

The TGF-β pathway plays a dual role within the gastric cancer immune microenvironment. Its Smad2/3-dependent pathway suppresses M1-associated inflammatory responses while concurrently upregulating M2 markers such as Arg1 and CD206 [31]. Meanwhile, non-canonical pathways participate in the regulation of cellular metabolism via PI3K/AKT and MAPK signaling [32]. Recent transcriptomic analyses reveal a strong correlation between TGF-β activation and M2 metabolic reprogramming, suggesting a bridging role for TGF-β between energy homeostasis and immune function [33].

The PI3K/AKT/mTOR pathway serves as a “metabolic-transcriptional” hub for regulating macrophage polarization, driving the transition toward the pro-tumorigenic M2 phenotype (tumor-associated macrophages, TAMs) through the induction of critical metabolic reprogramming [34]. Within the gastric cancer microenvironment, activation of this pathway inhibits the glycolysis pathway characteristic of M1 macrophages, instead promoting fatty acid oxidation (FAO) and histone acetylation via the activation of the Akt-Acly axis, thereby initiating the transcription of M2-related genes at the epigenetic level [35]. Concurrently, the mTOR signaling axis synergistically upregulates the expression of core transcription factors, including PPARγ, HIF-2α, and STAT6, significantly enhancing the synthesis of immunosuppressive molecules such as Arg-1 and CD206 [36]. This PI3K/AKT/mTOR-mediated phenotypic switch not only establishes an immune evasion “barrier” in gastric cancer but also significantly promotes clinical progression and poor prognosis by secreting pro-angiogenic factors and inducing chemoresistance. Table 1 summarizes the key molecular drivers regulating macrophage polarization in the gastric cancer microenvironment and their respective roles.

**Table 1:** Core signaling pathways and molecular mechanisms of macrophage polarization in gastric cancer

Signaling Pathway	Stimuli/Regulators	Polarization	Target markers	Mechanism and impact	Clinical relevance	Evidence level	References
STAT1	IFN-γ	M1	IL-12, TNF-α, iNOS	Promotes anti-tumor activity and inflammatory response	Low expression correlates with poor prognosis in liver metastasis	In vitro + Clinical	[37]
STAT3	IL-6, CSF-1, EGF	M2	IL-10, TGF-β, Arg1	Induces immune tolerance and angiogenesis; linked to clinical metastasis	High activation associated with peritoneal dissemination	In vitro + In vivo + Clinical	[28, 29]
NF-κB	LPS, IFN-γ, IL-6	M1/M2	p65, p50, inflammatory cytokines	Intensity-dependent: short-term leads to M1; chronic low-level leads to M2	Sustained activation correlates with advanced stage	In vitro + In vivo	[30]
TGF-β/Smad	TGF-β1, TGF-β2	M2	Smad2/3, Arg1, CD206	Inhibits M1 response; couples energy homeostasis with immune function	High expression associated with liver metastasis recurrence	In vitro + Clinical	[31–33]
PI3K/AKT/mTOR	Lactate, exosomal miR-21	M2	HIF-2α, PPARγ	Regulates metabolic state; promotes lipid oxidation over glycolysis	Activation correlates with chemoresistance	In vitro + In vivo	[34–36]

It is essential to emphasize that the aforementioned signaling pathways do not operate in isolation; instead, they intersect to form a synergistic “immunosuppressive network”. For instance, high levels of lactate within the gastric cancer microenvironment act as a metabolic signal that activates the PI3K/AKT/mTOR pathway, promoting lipid oxidation to sustain the M2 phenotype. Simultaneously, the resulting acidic microenvironment can trigger the release and activation of TGF-β, further reinforcing Smad-mediated polarization. This interconnected regulatory pattern not only endows TAMs with remarkable phenotypic stability but also poses a significant challenge for reversing polarization through single-target interventions.

### Cross-regulation and feedback mechanisms among signaling pathways

Different signaling pathways form a multi-layered cross-talk network during the process of macrophage polarization, collectively reinforcing immunosuppressive homeostasis. First, a bidirectional regulatory relationship exists between the STAT and NF-κB pathways. STAT1 and NF-κB can synergistically induce the transcription of pro-inflammatory genes, thereby sustaining M1-type functionality [38]. Conversely, the hyperactivation of STAT3 inhibits the nuclear translocation of NF-κB, thereby attenuating pro-inflammatory responses and facilitating M2 polarization [39]. Meanwhile, NF-κB-mediated IL-6 secretion can positively activate STAT3, forming an “IL-6/STAT3/NF-κB” pro-tumor positive feedback loop [40], which serves as a key driver of immune evasion in gastric cancer.

The TGF-β and PI3K/AKT/mTOR pathways exhibit close synergy in metabolic regulation. TGF-β not only activates PI3K/AKT via non-canonical pathways but also induces mTOR signaling to sustain mitochondrial function, thereby stabilizing the M2 phenotype [36]. Reciprocally, mTOR activation enhances the transcriptional response of TGF-β, establishing a metabolic-signaling cyclic amplification effect [41]. Recent multi-omics studies have further revealed that lactate-mediated mTOR activation synergizes with the TGF-β pathway to reinforce the immunosuppressive network and promote metastasis formation [42]. This suggests that simultaneously targeting both metabolic and signaling nodes within this network represents an effective strategy for combined intervention.

The non-coding RNA (ncRNA) regulatory network acts as a central integration node for the crosstalk among multiple signaling pathways, mediating signal transduction and functional interplay between distinct pathways to orchestrate the precise regulation of downstream biological effects. Specifically, gastric cancer cell-derived exosomal miR-21 [43], miR-146b [44], and lncRNA HOTAIR [45] have been shown to simultaneously target molecules such as PTEN and SOCS1/3. This action relieves the inhibition of the PI3K/AKT and STAT3 pathways, thereby achieving cross-pathway synergistic activation. This mechanism has been validated in both gastric cancer patient tissue samples and in vitro co-culture models, highlighting the critical role of ncRNAs in signal integration and immune homeostasis [46].

## Bidirectional interaction mechanisms between gastric cancer cells and macrophages

The bidirectional interaction between gastric cancer cells and tumor-associated macrophages (TAMs) constitutes a critical mechanism driving the remodeling of the tumor immune microenvironment and the formation of distant metastasis. This interaction is characterized by a continuous dynamic process involving signal induction, functional remodeling, and metabolic coupling.

Initially, gastric cancer cells drive the polarization of monocytes toward a pro-tumorigenic M2 phenotype through the secretion of cytokines and exosomes. Existing studies indicate that CSF-1 [47], IL-6 [48], VEGF [49], and TGF-β [50] secreted by gastric cancer cells activate the STAT3, Smad2/3, and PI3K/AKT signaling pathways. This prompts macrophages to upregulate CD206, Arg1, and IL-10, thereby establishing an immunosuppressive microenvironment. Furthermore, tumor-derived exosomes enriched with non-coding RNAs such as miR-205 [51], miR-25—3p [52], and circNEIL3 [53] can downregulate PTEN and SOCS1/3. This action sustains the activity of PI3K/AKT/mTOR signaling, induces metabolic reprogramming in macrophages, and stabilizes the M2 phenotype. Notably, lactate generated by the high glycolytic activity of tumor cells further activates the mTOR pathway via the GPR81 receptor, enhancing M2 polarization and immunosuppressive functions; this reciprocal mechanism between metabolic signaling and polarization pathways has been validated at the single-cell level.

Polarized M2 macrophages reciprocally regulate the malignant phenotype of gastric cancer cells through multifaceted pathways. TGF-β [54] and IL-10 [55] secreted by M2 macrophages induce epithelial-mesenchymal transition (EMT) in gastric cancer cells by upregulating the expression of Snail, N-cadherin, and Vimentin, thereby attenuating cellular adhesion and enhancing invasiveness. Concurrently, the release of VEGF and MMP9 promotes angiogenesis and matrix degradation, providing structural pathways for tumor cell intravasation and metastasis [55, 56]. Furthermore, M2-derived extracellular vesicles (M2-EVs) are enriched with miR-21 [57], miR-223, and S100A9; these bioactive cargoes activate PI3K/AKT [57], STAT3, and NF-κB signaling within gastric cancer cells, enhancing their proliferation, anti-apoptotic capabilities, and immune evasion potential. Animal model experiments further demonstrate that inhibiting the generation of M2-EVs or blocking their receptor recognition significantly reduces the rate of gastric cancer metastasis, suggesting that macrophage exosomes play a pivotal role in signal amplification and phenotype maintenance.

Finally, gastric cancer cells and macrophages establish a closed loop of mutual energy supply and signaling feedback at the metabolic level. The acidic microenvironment resulting from lactate efflux by tumor cells induces a metabolic shift in macrophages from glycolysis to oxidative phosphorylation and activates mTOR to maintain their antioxidant metabolic state [58]. Reciprocally, fatty acids, cholesterol, and glutamine secreted by M2 macrophages are directly taken up by gastric cancer cells to replenish lipid and amino acid metabolic pathways, thereby supporting their high energy demands and sustained proliferation [59]. Concurrently, metabolites and transcriptional signaling loops form a bidirectional positive feedback circuit involving PI3K/AKT-mTOR and STAT3, facilitating the synergistic coexistence of immunosuppression and energy supply, which further reinforces the homeostasis of the metastatic microenvironment [60]. Analyses using single-cell transcriptomics and spatial metabolomics have confirmed the existence of significant metabolic symbiotic characteristics between M2 macrophages and cancer cells within gastric cancer liver metastases and peritoneal dissemination regions [61]. To clearly elucidate the bidirectional communication between macrophages and gastric cancer cells mediated by exosomes and its regulatory role in the tumor microenvironment, the relevant mechanisms are summarized in Fig. 2.

**Fig. 2:**
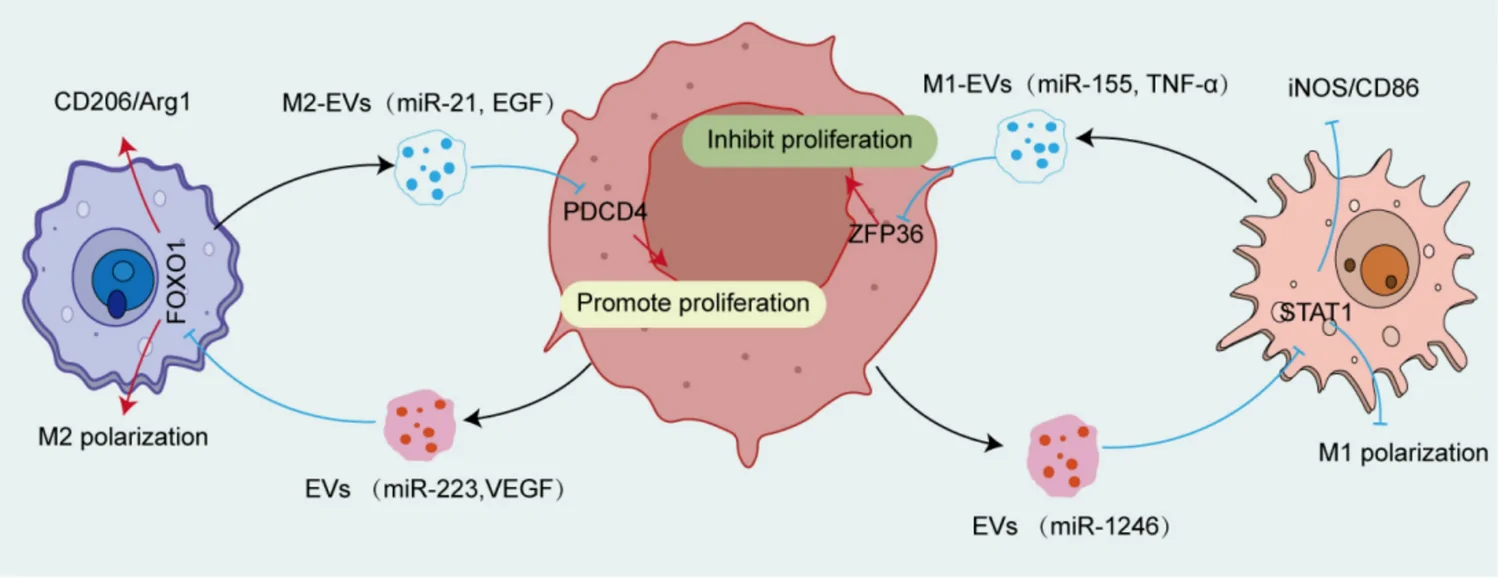
Bidirectional communication between macrophages and gastric cancer cells mediated by extracellular vesicles (EVs) and their regulatory role in the tumor microenvironment

## Clinical applications and therapeutic strategies

The mechanisms underlying macrophage polarization provide a novel theoretical foundation for targeted intervention in metastatic gastric cancer. Current translational research primarily focuses on two core objectives: first, inhibiting the recruitment and survival of pro-tumorigenic macrophages; and second, reprogramming the pro-tumorigenic phenotype toward an anti-tumorigenic phenotype. By combining these strategies with immune checkpoint inhibitors, research aims to overcome immune resistance in gastric cancer and ultimately improve patient prognosis.

Therapeutic strategies targeting tumor-associated macrophages (TAMs) are primarily categorized into two approaches: blocking the recruitment and survival of TAMs, and reprogramming their polarization state. Regarding the inhibition of TAM recruitment, the CSF1/CSF1R signaling axis represents the most extensively studied target. In gastric cancer, CSF1R is expressed on TAMs, and activation of its signaling pathway promotes M2 macrophage polarization, thereby supporting tumor growth and metastasis [47]. However, direct evidence from preclinical studies of CSF1R inhibitors specifically in gastric cancer models remains extremely limited; current mechanistic understanding is largely extrapolated from other gastrointestinal malignancies, such as esophageal adenocarcinoma and colorectal cancer. In esophageal adenocarcinoma models, pexidartinib (PLX3397) combined with PD-1 inhibitors has been shown to reduce the infiltration of M2-type TAMs and induce their reprogramming toward the M1 phenotype, thereby overcoming PD-1 resistance [62], suggesting potential efficacy for this strategy in gastric cancer. Nevertheless, in gastric cancer-specific clinical trials, definitive efficacy data (e.g., ORR, DCR) for CSF1R inhibitors as monotherapy or in combination regimens have not yet been disclosed, and their clinical effectiveness remains unknown.

The CCL2-CCR2 axis is another critical target for regulating monocyte recruitment. In preclinical studies of gastric cancer, CCR2 antagonists theoretically block CCL2-mediated TAM recruitment, thereby inhibiting tumor invasion and metastasis; however, there is currently a lack of gastric cancer-specific preclinical validation and clinical trial data [63]. Current mechanistic understanding is primarily based on other malignancies: in clinical trials for pancreatic and colorectal cancer, the dual CCR2/5 antagonist BMS-813160 combined with chemotherapy or immune checkpoint inhibitors has demonstrated some anti-tumor activity [64]. Nevertheless, these trials explicitly excluded gastric cancer patients and did not include a gastric cancer cohort.

CD47 serves as a critical antiphagocytic signal overexpressed on the surface of gastric cancer cells, effectively suppressing macrophage-mediated phagocytosis. In preclinical gastric cancer models, disrupting the CD47–SIRPα axis using agents such as SIRPα-Fc fusion proteins has been shown to enhance the phagocytic activity of macrophages against patient-derived cells (PDCs) and restrict tumor progression in humanized PDX models [65]. Clinical evidence for this strategy is supported by a Phase I trial (NCT03013218) evaluating the CD47-blocking antibody ALX148 combined with trastuzumab in HER2-positive gastric cancer, which yielded an objective response rate (ORR) of 21.1% and a disease control rate (DCR) of 26.3% [66]. While these findings provide preliminary validation for the feasibility of CD47-targeted therapy in gastric cancer, the limited sample size and the nature of the combination regimen necessitate further investigation to confirm the efficacy of monotherapy and achieve large-scale clinical validation.

Reprogramming TAMs from a pro-tumorigenic to an anti-tumorigenic phenotype represents another core therapeutic strategy. Regarding pan-cancer mechanisms, STAT3 inhibitors (such as corosolic acid) can suppress M2-like polarization by inhibiting the STAT3 signaling pathway [67]. In head and neck squamous cell carcinoma, PI3Kγ inhibitors have been shown to promote the differentiation of macrophages toward the M1 phenotype [68]. Similarly, TLR agonists can induce M1 polarization and enhance anti-tumor immune responses in breast cancer [69]. However, there is currently a lack of experimental validation specifically focused on gastric cancer.

Given the limited efficacy of monotherapy, combination therapy has become the primary focus of translational research. Theoretically, combining CSF1R inhibitors with PD-1/PD-L1 inhibitors holds synergistic potential in gastric cancer by depleting M2-type TAMs and increasing T-cell infiltration to restore anti-tumor immunity. However, to date, there are no published reports of clinical trials specifically evaluating CSF1R inhibitors combined with immune checkpoint inhibitors in gastric cancer; this synergistic effect has only been preliminarily validated in esophageal adenocarcinoma models [70], and clinical translation to gastric cancer requires further disease-specific trial evidence. Chimeric antigen receptor-macrophage (CAR-M) therapy represents an emerging cellular immunotherapy. Preclinical studies indicate that CAR-M cells can specifically phagocytose tumor cells and activate local anti-tumor immunity [71]; however, its application in solid tumors remains in the preclinical or Phase I clinical stages. For instance, the first CAR-M therapy, CT-0508 (NCT04660929), targets HER2-positive solid tumors in general, and clinical data specific to gastric cancer are currently unavailable [72].

Despite a robust preclinical foundation, most TAM-targeted strategies have failed to achieve the expected outcomes in the clinical treatment of gastric cancer, primarily due to several critical factors. First, TAMs exhibit high functional heterogeneity and plasticity, particularly across different metastatic sites. In gastric cancer patients with peritoneal metastasis, M2-type TAMs are significantly increased, and the TAM phenotype varies significantly depending on histological type, anatomical location, and disease stage [73]. Furthermore, the immune landscape of metastatic lesions across different pathological subtypes demonstrates substantial spatiotemporal heterogeneity. Specifically, a “pre-metastatic niche” characterized by tissue fibrosis, inflammatory infiltration, and an immunosuppressive microenvironment dominated by M2-type TAMs is established prior to peritoneal metastasis; this spatial heterogeneity makes it difficult for single-target interventions to fully abrogate pro-tumorigenic effects [74].

Second, the tumor microenvironment possesses significant pathway redundancy. Following CSF1R inhibition, TAMs can regain immunosuppressive functions through alternative ligands such as IL-34 [75], reactivation of the JAK2/STAT3 pathway, or TGF-β induction [76]; simultaneously, the compensatory expansion of myeloid-derived suppressor cells (MDSCs) may reconstitute the immunosuppressive environment [77]. Systemic toxicity also remains a major constraint, as CSF1R inhibitors are associated with anemia, leukopenia, and hepatotoxicity [78]. Moreover, the systemic depletion of tissue-resident macrophages, such as Kupffer cells, may disrupt normal immune homeostasis and increase infection risks, severely limiting clinical dosing [79]. Finally, the lack of validated biomarkers for patient stratification—such as TAM infiltration density [80], M2 polarization status [81], or alternative ligand expression (e.g., IL-34) [82]—remains in the early validation stage, hindering the implementation of precision therapy and diminishing overall clinical benefit.

Although many TAM-targeted strategies are currently extrapolated from other malignancies, their preliminary progress in related fields provides a critical reference for clinical translation in gastric cancer, as summarized in Table 2.

**Table 2:** Summary of therapeutic strategies targeting tumor-associated macrophages (TAMs) in gastric cancer and other malignancies

Strategy	Target	Clinical phase/research status	Cancer type(s)	Key outcomes/limitations	References
Blocking recruitment and survival	CSF1/CSF1R	Phase I/II	Esophageal adenocarcinoma, colorectal cancer	Combined with PD-1 inhibitors to overcome resistance and reduce M2-TAMs; lack of specific clinical data in gastric cancer	[62]
	CCL2/CCR2	Phase I/II	Pancreatic and colorectal cancer	Dual CCR2/5 antagonist (BMS-813160) showed activity in combination; gastric cancer cohorts were excluded	[64]
Phagocytosis enhancement	CD47/SIRPα	Phase I (NCT03013218)	HER2+ gastric cancer	ALX148 + Trastuzumab: ORR 21.1%, DCR 26.3%; small sample size; monotherapy efficacy unconfirmed	[66]
Phenotypic reprogramming	STAT3	Preclinical	Pan-cancer	Corosolic acid inhibits M2-like polarization; Lacks gastric cancer-specific validation	[67]
	PI3Kγ	Preclinical	HNSCC	Promotes M1 differentiation; lacks gastric cancer-specific validation	[68]
	TLR Agonists	Preclinical	Breast cancer	Induces M1 polarization and enhances anti-tumor immunity; lacks gastric cancer-specific validation	[69]
Cellular immunotherapy	CAR-M	Phase I (NCT04660929)	HER2+ solid tumors	Targeted phagocytosis and activation of local immunity; no gastric cancer-specific clinical data reported	[71, 72]

## Conclusion and outlook

The polarization of tumor-associated macrophages (TAMs) is a core mechanism driving distant metastasis and immune evasion in gastric cancer. Existing research evidence indicates that M1-type macrophages mediate anti-tumor inflammatory responses, whereas M2-type macrophages profoundly influence the invasive behavior of gastric cancer by remodeling the immunosuppressive microenvironment and promoting angiogenesis and the formation of the metastatic niche. Core signaling pathways, including STAT3, NF-κB, TGF-β, and PI3K/AKT, not only constitute the molecular foundation of polarization but also form a complex regulatory network. Macrophage polarization has evolved from a simple immune phenomenon into a regulatory hub that actively participates in tumor evolution, providing critical theoretical support for elucidating the immunological essence of gastric cancer metastasis.

Although mechanistic research has made significant progress, translating these findings into clinical applications still faces numerous challenges. The primary bottleneck at present lies in the ambiguity of the M1/M2 phenotypic boundaries; they are not static binary opposites but rather a continuum dynamically regulated by spatiotemporal factors, which increases the complexity of intervention strategies. Furthermore, the analysis of a single pathway is insufficient to cover the overall heterogeneity of the tumor microenvironment, and the significant differences between animal models and human samples limit the translational efficiency of experimental results. Therefore, future research urgently needs to utilize single-cell multi-omics, spatial transcriptomics, and multi-dimensional immune analysis technologies to construct high-precision spatiotemporal dynamic models, thereby revealing the true functional characteristics of macrophages in human metastatic lesions.

Looking forward, translational research on TAM-targeted therapies should focus on several critical directions. First, it is essential to conduct gastric cancer-specific clinical trials to reduce over-reliance on evidence from other malignancies and obtain more reliable, disease-specific clinical data. Second, precision treatment strategies guided by biomarkers must be established to identify sensitive patient populations based on TAM infiltration density, polarization states, and signaling pathway activation. Third, rational combination regimens should be optimized, integrating TAM-targeted agents with immune checkpoint inhibitors, chemotherapy, or anti-angiogenic drugs to achieve synergistic effects. Finally, the development of more efficient and tumor-targeted drug delivery systems is necessary to mitigate systemic toxicity, thereby enhancing both safety and clinical efficacy.

## Data Availability

Not applicable.
